# Hydroxy-γ-sanshool from *Zanthoxylum bungeanum* (prickly ash) induces apoptosis of human colorectal cancer cell by activating P53 and Caspase 8

**DOI:** 10.3389/fnut.2022.914638

**Published:** 2022-08-01

**Authors:** Chen Zhaojun, Tan Lulin, Feng Xin, Singab Abdel-nasser, Lei Zunguo, Liu Xiong

**Affiliations:** ^1^College of Food Science, Southwest University, Chongqing, China; ^2^Guizhou Provincial Academy of Agricultural Sciences, Guiyang, China; ^3^Department of Pharmacognosy, Ain Shams University, Cairo, Egypt

**Keywords:** hydroxy-γ-sanshool, *Zanthoxylum bungeanum*, apoptosis, cell arrest, colorectal cancer cells

## Abstract

Sanshools, long-chain polyunsaturated amides in *Zanthoxylum bungeanum* (prickly ash), have important bioactivity. The objective was to assess inhibitory effects and molecular mechanisms of sanshools isolated from supercritical fluid (SCF) extract on human colon adenocarcinoma cells (HCT-116) cultured *in vitro*. Cells were exposed to various concentrations (0, 50, 90, or 130 μM) of sanshools for 24 or 48 h, with assessment of apoptosis and cell cycle arrest as well as regulatory gene and protein expression associated with apoptosis and the cell cycle. Sanshools profoundly inhibited growth of HCT-116 cells, with hydroxy-γ-sanshool (HRS) being the optimal active component (IC_50_ = 88.01 μM) inhibiting cell proliferation and having no cytotoxic effect to normal cells (IC_50_ = 481.52 μM) by CCK-8 assay. In HCT-116 cells, HRS inhibited cell growth, induced morphological distortion, and arrested the cell cycle at G1 phase (50.31 ± 4.13% vs. 72.16 ± 8.14% in Control and 130 μM HRS, respectively), and also caused programmed cell death in a dose-dependent manner. The percentage of apoptotic cells were remarkably increased after treated with HRS (6.2, 11.9, 19.8, and 30.7% for 0, 50, 90, and 130 μM, respectively). Moreover, in HCT-116 cells, HRS significantly inhibited mRNA and protein levels of Cyclin D1, CDK4, PCNA, and increased mRNA and protein levels of P21, P53, Fas, and Caspase 8. Furthermore, inhibitors of P53 and Caspase 8 proteins significantly mitigated the HRS-induced cell cycle arrest and apoptosis. In conclusion, our study provides evidence that HRS induced human colorectal cancer cell apoptosis by up-regulating P53 and Caspase 8.

## Introduction

Colorectal cancer (CRC), a malignant tumor on the wall of the rectum or colon, ranks as the second leading cause of cancer incidence and the third most common cancer worldwide (1). Currently, the dominant and most effective treatment strategy is pharmacotherapy with 5-fluorouracil, leucovorin, irinotecan, and oxaliplatin (2). However, these compounds have many side effects and disadvantages, including bone marrow suppression, gastrointestinal tract lesions, hair loss, nausea, high cost, and development of clinical resistance (3). Therefore, there is an impetus to search for new compounds with low toxicity that could safely inhibit CRC development.

Prevention and treatment of CRC can be done with several natural bioactive compounds derived from plants and microorganisms, including quercetin, kaempferol, phenolic acids, ellagic acid, and protocatechuic acid (3, 4). Underlying mechanisms may be activation-induced apoptosis and cell cycle arrest (5), through cell signaling pathways (NF-κB, Wnt/β-catenin, ERK/MAPK, and PI3K/Akt/mTOR) (6). Apoptosis is mediated through three crucial pathways: intrinsic mitochondrial pathway, extrinsic death receptor pathway, and intrinsic endoplasmic reticulum pathway. Caspase 9 is the intrinsic pathway whereas Caspase 8 is the extrinsic pathway; both pathways converge to Caspase 3, causing breakdown of DNA and proteins, resulting in phagocytosis by macrophages.

The cell cycle is a complex process in growth and proliferation of cancer cells, involving numerous regulatory proteins, including cdks and cyclin (6). Sequential activation of cyclin-dependent kinases and phosphorylation of critical substrates facilitates orderly progression of the cell cycle (7, 8). Furthermore, complexes formed by CDK4 and D-type cyclins have been strongly implicated in the control of cell proliferation during the G1 phase (9, 10).

Prickly ash, called “Huajiao” in China, is mainly distributed in Asia, America, Oceania, and Africa. This plant belongs to the family Rutaceae and genus Zanthoxylum (11, 12), and is used as medicine, spice, or woody oil. Reported bioactivities of Zanthoxylum-based extracts include antioxidant, antitumor, antibacterial, anti-inflammatory, and hypoglycemic effects (11). Sanshools, the dominant sector of polyunsaturated amides (13) characterized by three conjugated double bonds, have abundant pharmacological effects, including unique tingling and numbing. Yields, species, and biological activities of sanshools are affected by extraction (13, 14). Sanshools regulated blood glucose (15) and serum lipid (15) concentrations and induced apoptosis in hepatoma cells (16) and other human cancer cell lines including DLD-1, HepG2, and Caco-2 (12). However, sanshool-induced apoptosis has not been well characterized. In addition, there are >10 kinds of sanshools (11) with various structural properties having distinct physiological activities. Therefore, there is a compelling need to explore the structure-activity relationship of various sanshools.

Objectives were to compare induced apoptosis effects of three sanshools derived from one species of prickly ash and explore mechanisms promoting CRC apoptosis.

## Materials and methods

### Chemicals

Supercritical fluid (SCF) extract of *Zanthoxylum bungeanum* was purchased from Zhengzhou Xuemailong Food Spice Co., Ltd. in Henan Province, China. Both HCT-116 (human colon adenocarcinoma cell line), and HEK293T cells were obtained from the National Collection of Authenticated Cell Cultures in Shanghai, China. McCoy's 5A media, DMEM media, fetal bovine serum (FBS), and 0.25% trypsin solution were obtained from Invitrogen (Carlsbad, CA, USA). The Caspase 8 inhibitor Z-IETD-FMK and P53 inhibitor PFTα hydrobromide were obtained from MedChemExpress (Monmouth Junction, NJ, USA). Primary antibodies (anti-CDK4, anti-cyclin D1, anti-PCNA, anti-BAX, anti-Caspase 3, anti-Cleaved Caspase 3, anti-P53, anti-Bcl-2, anti-Caspase 9, anti-Cleaved Caspase 9, anti-Fas, anti-P21, anti-Caspase 8, anti-Cleaved Caspase 8, and anti-β-actin), anti-mouse, and anti-rabbit secondary were obtained from the Beyotime Institute of Biotechnology (Shanghai, China).

### Sample preparation

Sanshools were extracted as described (15), with a minor modification. The SCF extracts (60 g) were mixed with water-free silica (120 g), and thrice defatted with 400 mL anhydrous ether. The defatted sample was homogenized with 200 mL methanol at 55°C for 6 h; the crude extract was refluxed with 60 mL petroleum ether overnight after filtering and vacuum evaporation. The supernatant was collected and crystallized at −20°C. The suspension was dried using dry N_2_ gas at 4°C.

Further purification was done by semi-preparative HPLC (Waters Technologies, Milford, MA, USA). The mobile phase consisted of (A) water and (B) acetonitrile. A Waters preparative C18 column (9.4 ×250 mm, 5 μm) was used with the following gradient elution program: 0 min, 35% B; 30 min, 75% B; 40 min, 100% B; 45 min, 100% B; and 50 min, 35% B. Purified extract was subjected to mass spectrometry using an API 4000 QTrap mass spectrometer (AB/Sciex, Framingham, MA, USA) fitted with an electrospray ionization (ESI) source, using the following parameters: turbo ion spray probe, 400°C, ion spray voltage, 4500 V, entrance potential, 6 V, and collision energy of 10 V. In addition, nuclear magnetic resonance (NMR) including ^13^C NMR and ^1^H NMR was done as described 24 with a VNMRS600 NMR spectrometer (Agilent, Palo Alto, USA) to identify the purified extract.

### Cell proliferation assay

Inhibition of growth of HCT-116 cells was assessed using the Cell Counting Kit8 (APExBIO, Houston, TX, USA). Briefly, 96-well plates were plated with 5 × 10^3^ cells per well and incubated for 12 h. Then, cells were treated with HRS (0, 30, 50, 70, 90, 110, 130, 150, or 170 μM) for 24 h. Thereafter, 10 μL of CCK8 reagent was added and incubated at 37°C for 2 h. Absorbance was measured by a microplate reader at a wavelength of 450 nm. Inhibition effects of HRS on HCT-116 were calculated by comparison to a control group.

### Morphological assessment

Morphological changes in HCT-116 cells and HEK293T cells treated with HRS (0, 50, 90, or 130 μM) for 12 or 24 h were observed using an inverted optical microscope at 200 × magnification (Nikon 80i, Tokyo, Japan).

### Cell cycle analysis

The HCT-116 cells and HEK293T cells with a density of 3 × 10^5^ cells/well were exposed to HRS (0, 50, 90, or 130 μM) for 24 h at 37°C. Then, cells were treated with trypsin, gathered, and fixed with 5 mL 80% ethanol at 4°C for 18 h, followed by washing with PBS. Propidium iodide (PI) was added and cells incubated for 30 min in the dark. Cell cycle distribution was assessed by flow cytometry (FACS Canto II plus, BD BioSciences, USA) with a cell cycle kit (BD, NJ, USA).

### Hoechst 33342 staining assay

Apoptosis staining was detected by Hoechst 33342 (Solarbio, Beijing, China). In brief, after treatment with HRS (0, 50, 90, or 130 μM) for 24 h, cells were stained with Hoechst 33342 staining solution for 20 min at 4°C. Then, cells were washed with PBS and observed with fluorescence microscopy at 200 × magnification (Nikon 80i, Tokyo, Japan).

### Apoptosis analysis

After treatment with a series of HRS solutions (0, 50, 90, or 130 μM) for 24 h, the HCT-116 cells were harvested. Washed cells were resuspended with 400 μL of 1 × Annexin V binding buffer at a final concentration of 3 × 10^5^ cells/mL. Then, 5 μL Annexin V-FITC (BD, New Jersey, USA) was added and incubated in the dark for 15 min at 2–8°C. Thereafter, 5 μL PI (BD, New Jersey, USA) was added to the mixture and incubated for 10 min. Cell apoptosis was examined using flow cytometry (FACS Canto II plus, BD BioSciences, USA).

### The mRNA extraction and quantitative real-time PCR

Cells (density, 3 × 10^5^ cells/well) were treated with HRS (0, 50, 90, or 130 μM) and cultured in a 6-well plate for 24 h. Total RNA was isolated by TRIzol (Takara, Dalian, China). Synthesis of cDNA was conducted by PrimeScript™ RT Reagent Kit (Thermo Fisher Scientific Inc., Waltham, MA, USA) and RT-PCR was done with an Applied Biosystems^®^ QuantStudio™ 5 Flex Real-Time PCR System (Thermo Fisher Scientific Inc.). The reaction conditions were: 2 min at 95°C, followed by 40 cycles of 15 s at 95°C, and 1 min at 60°C. Relative expression of the selected gene was calculated with the 2^−ΔΔCt^ method, with β-actin used as a control. Primer sequences are shown (Table 1).

**Table 1:** Primers used for real-time PCR.

**Gene**	**Direction**	**Sequence (5**′**-3**′**)**
Bcl-2	Forward	CGACTTCGCCGAGATGTCCAG
	Reverse	CAGGTGCCGGTTCAGGTACTCA
Bax	Forward	TTTGCTTCAGGGTTTCATCCAGG
	Reverse	TGAGACACTCGCTCAGCTTCTTG
Caspase 3	Forward	ACTGGACTGTGGCATTGAG
	Reverse	AACCAGGTGCTGTGGAGTA
Caspase 9	Forward	GAGATTCGCAAACCAGAGG
	Reverse	TCACGGCAGAAGTTCACAT
CDK2	Forward	GAAACAAGTTGACGGGAGA
	Reverse	AAGAGGAATGCCAGTGAGA
SCF	Forward	TTGGATAAGCGAGATGG
	Reverse	TTTCTTTCACGCACTCC
CDK4	Forward	GTTCGTGAGGTGGCTTTACTG
	Reverse	GTCCTTAGGTCCTGGTCTACATG
GSK3β	Forward	GTCAAGTAATCCACCTCTG
	Reverse	GTCTGTCCACGGTCTCC
PCNA	Forward	TAGTAAAGATGCCTTCTGGTGA
	Reverse	TATGGTAACAGCTTCCTCCTC
β-actin	Forward	CTGGGACGACATGGAGAAA
	Reverse	GCACAGCCTGGATAGCAAC
FAS-L	Forward	TTCTTCCCTGTCCAACC
	Reverse	AATCCTACCAAGGCAACC
BID	Forward	ACAACGGTTCCAGCCTCA
	Reverse	CATCGTAGCCCTCCCACT
PUMA	Forward	GGGAGGAGGAACAGTGGGC
	reverse	CAGGGTGTCAGGAGGTGGGAG
Fas	Forward	CCAAGAAGGGAAGGAGT
	Reverse	GGTGTTGCTGGTGAGTG
TRAIL	Forward	ACAGACCTGCGTGCTGA
	Reverse	GGTCCCAATAACTGTCATCT
TRAIL-R1	Forward	GTGGCTGTGCTGATTGTCT
	Reverse	GCGAGTCTGCGTTGCTC
cyclinD1	Forward	GTCGCTGGAGCCCGTGAAA
	Reverse	CGGATGGAGTTGTCGGTGTA
P53	Forward	GCGTGTTTGTGCCTGTCCT
	Reverse	TGCTCGCTTAGTGCTCCCT
P15	Forward	CGGCAGCGATGAGGGTCT
	Reverse	GCCTCCCGAAACGGTTGACT
p21	Forward	GGATGTCCGTCAGAACCCA
	Reverse	CCTGCCTCCTCCCAACTCA
cdc25A	Forward	ACTTCCTTTACCGTCTGTC
	Reverse	AAACCATTCGGAGTGCTAC
GADD45	Forward	GAGCAGAAGACCGAAAGCG
	Reverse	TTCGTCACCAGCACGCAGT
cytc	Forward	GTATTCCTGCGGGTGAT
	Reverse	CGTTCTTGCGGTTTCTT
SMAD4	Forward	TTTGATGTGCCATAGACAA
	Reverse	GACCAGCCACCTGAAGC
FADD	Forward	GAGAAGGCTGGCTCGTCA
	Reverse	GGAGGTAGATGCGTCTGAGTT
Caspase 8	Forward	GCTTTGACCACGACCTT
	Reverse	ATGATGCCCTTGTCTCC
DFF45	Forward	TGATAAGTCCCTGACACCA
	Reverse	AGCCAATGCCACAAACT
Smac	Forward	TCTACTTCCAGGCTGTTTA
	Reverse	CTGCGCCAGTTTGATAT
TGFβ	Forward	TGGCGATACCTCAGCAACC
	Reverse	AAGGCGAAAGCCCTCAAT

### Protein extraction and western blot

Western blot analysis was done as described (17). Treated cells were washed thrice with PBS, followed by scraping with ice-cold RIPA buffer containing 1 mM protease phosphatase inhibitor and protease inhibitor. Protein concentrations were determined with a BCA protein quantification kit (Solarbio, Beijing, China). Furthermore, after boiling for 10 min, supernatant proteins were separated electrophoretically *via* SDS-PAGE and placed onto polyvinylide fluoride (PVDF) membranes. These membranes were blocked with 5% dried skim milk containing 0.1% Tween-20 (PBST) for 2 h at 37°C. The membrane was then incubated overnight at 4°C with primary antibodies. Finally, membranes were treated with a secondary antibody for 1 h at 37°C. Visualization was done with a High-sensitivity Chemiluminescence imaging system ChemiDocXRS+ (Bio-Rad, California, USA).

### Inhibition of caspase 8 and p53

Inhibitors were used as described (18, 19), with a minor modification. After exposure to 90 μM HRS for 22 h, HCT-116 cells were treated with P53 inhibitor (Pifithrin-α hydrobromide) (30 μM) or with Caspase 8 inhibitor (40 μM) (Z-IETD-FMK) for 2 h. Thereafter, analysis was done with Western blotting using p53 and Caspase 8 antibodies.

### Statistical analyses

All dates are expressed as mean ± standard deviation (SD) (*n* ≥ 3). One-way ANOVA and Student's *t*-test were used to detect the differences between various treatments. All analyses were done with SPSS17 (SPSS Inc., Chicago, IL, USA) and *p* < 0.05 was considered significant.

## Results

### HRS extraction from supercritical fluid extract

**Figure 1:**
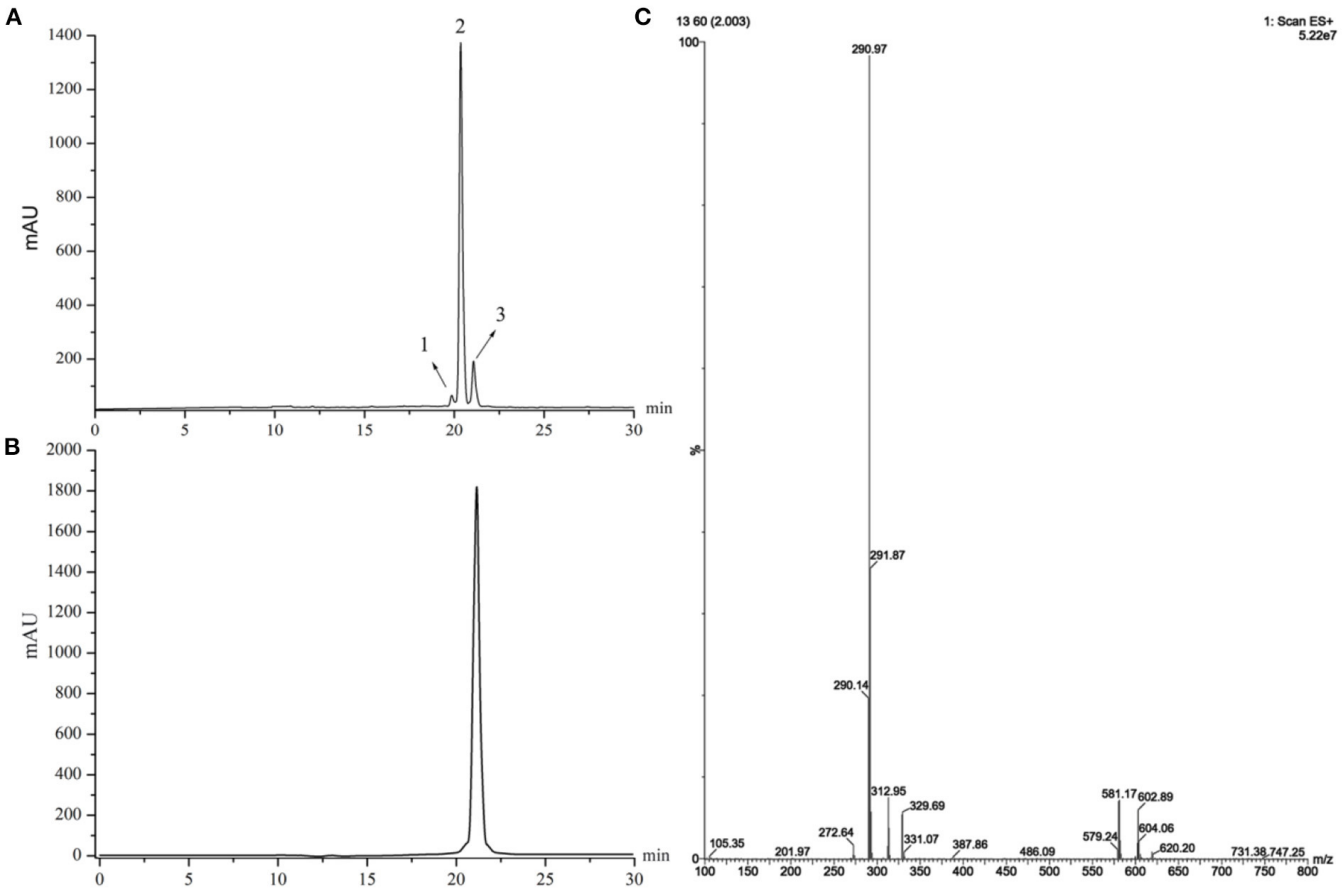
**(A)** HPLC chromatograms of a supercritical fluid extracted from *Zanthoxylum bungeanum*. **(B)** HPLC chromatograms of Compound 3. **(C)** MS spectra of Compound 3.

**Figure 2:**
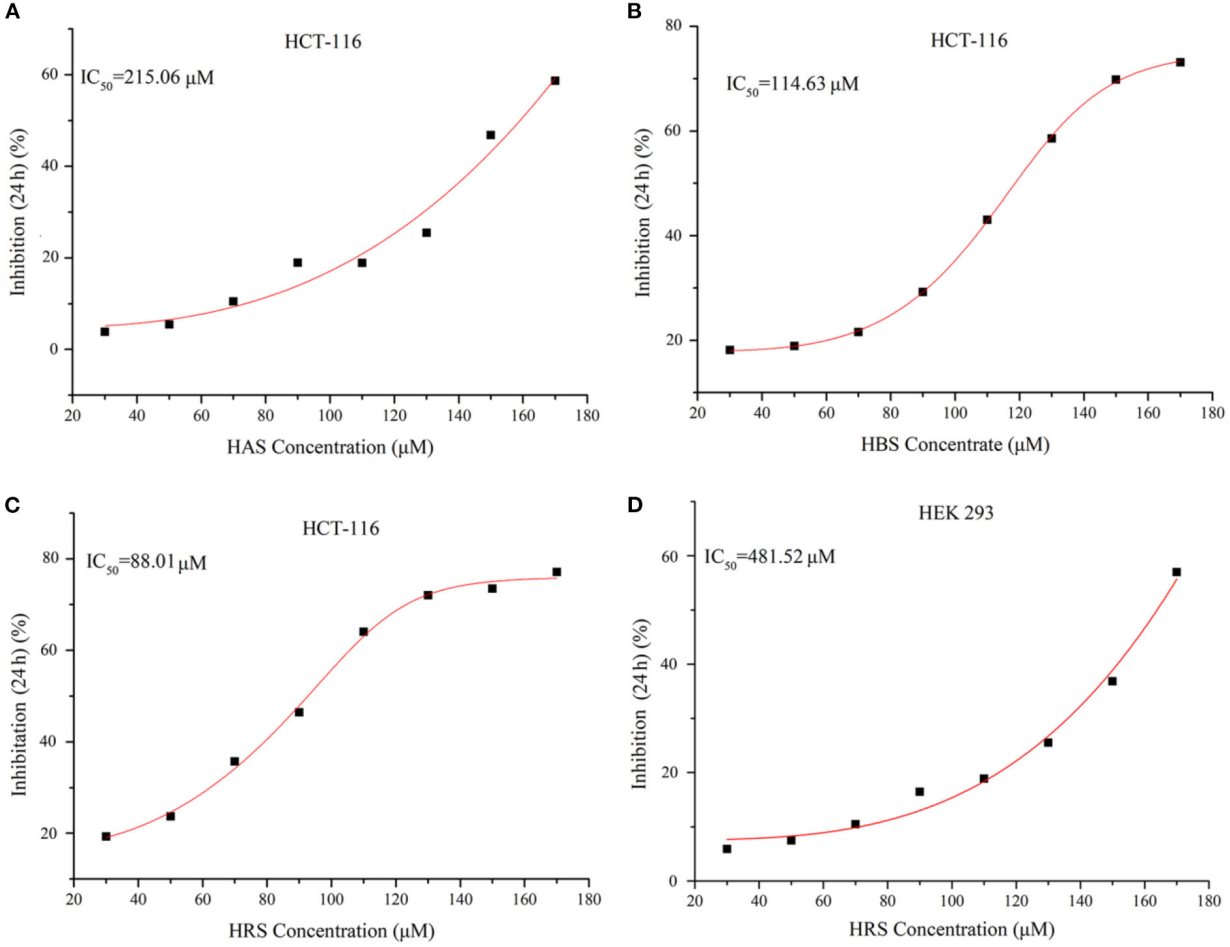
**(A)** Cell growth inhibition curves of Compound 1 (HAS) on HCT-116 cells. **(B)** Cell growth inhibition curves of Compound 2 (HBS) on HCT-116 cells. **(C)** Cell growth inhibition curves of Compound 3 (HRS) on HCT-116 cells. **(D)** Cell growth Inhibition curves of Compound 3 (HRS) on HEK293 cells (normal cells).

**Figure 3:**
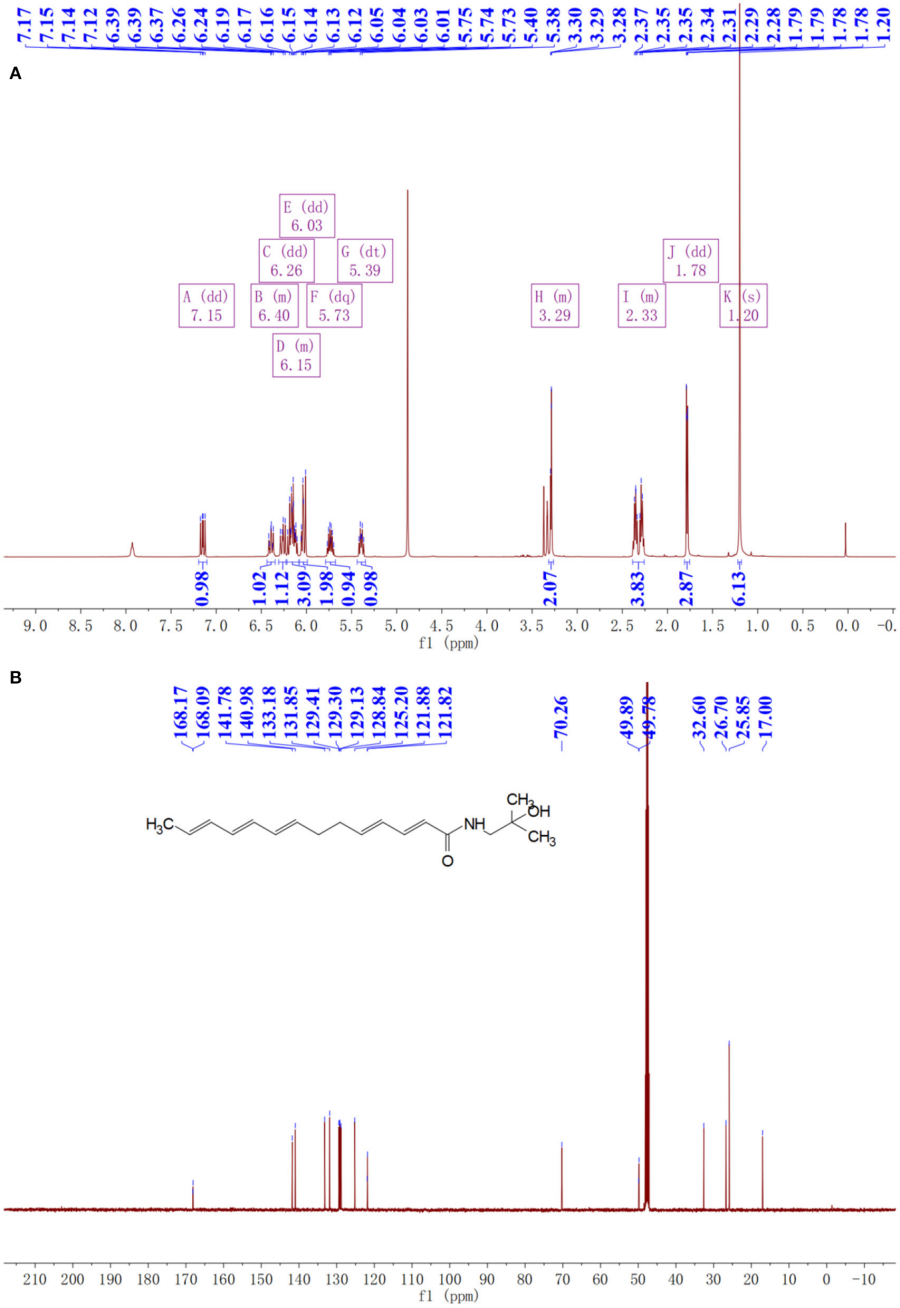
NMR spectra of prepared hydroxy-γ-sanshool (HRS). **(A)** ^1^H NMR spectra. **(B)** ^1^C NMR spectra.

The purity of extracted sanshools was reported as 93.42%. Peaks 1, 2, and 3 were situated at 19.825, 20.353, and 21.069, respectively (Figure 1A), and isolated by semi-preparative HPLC into three compounds to perform the cell inhibition assay. The purity of HAS, HBS, and HRS were determined as 95.32, 96.15, and 96.36%. Inhibitory effects of the three compounds on HCT-116 cell proliferation were concentration-dependent (Figures 2A–C). Compound 3 had the lowest IC_50_ value (88.01 μM) for HCT-116 cell lines compared to the other two compounds, whereas the IC_50_ for HEK293T was 481.52 μM (Figure 2D), indicating it was non-toxic to normal cell lines. Compound 3 was then identified by HPLC (Figure 1B) and MS, revealing an [M+H] ^+^ ion at m/z 290.97 (Figure 1C), with further identification by NMR in Figures 3A,B. ^1^H NMR (500 MHz, Methanol-*d*_4_) δ: 7.15 (dd, *J* = 15.1, 10.8 Hz, 1H), 6.44–6.35 (m, 1H), 6.26 (dd, *J* = 15.2, 10.7 Hz, 1H), 6.23–6.08 (m, 3H), 6.03 (dd, *J* = 12.9, 9.7 Hz, 2H), 5.73 (dq, *J* = 13.9, 6.9 Hz, 1H), 5.39 (dt, *J* = 10.9, 7.5 Hz, 1H), 3.32–3.26 (m, 2H), 2.39–2.26 (m, 4H), 1.78 (dd, *J* = 6.6, 1.3 Hz, 3H), 1.20 (s, 6H).

^13^C NMR (126 MHz, MeOD) δ: 168.17, 168.09, 141.78, 140.98, 133.18, 131.85, 129.41, 129.30, 129.13, 128.84, 125.20, 121.88, 121.82, 70.26, 49.89, 49.78, 32.60, 26.70, 25.85, 17.00. Compound 3 was identified as hydroxy-γ-sanshool based on the analysis of MS and NMR as well as a previous report (20).

### Effects of HRS on morphology of HCT-116 cells

**Figure 4:**
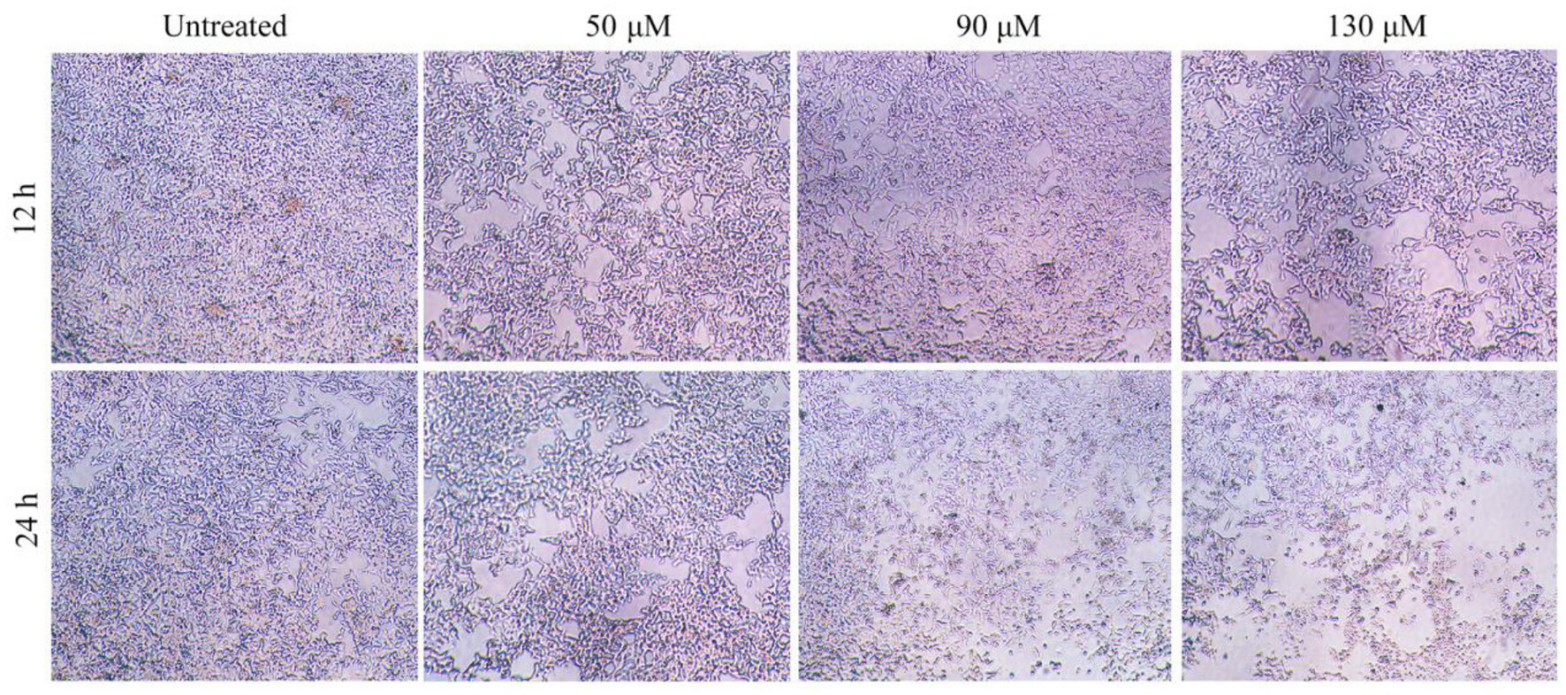
Effects of hydroxy-γ-sanshool (HRS) on morphological changes in HCT-116 cells. The original magnification was × 200.

Effect of HRS on the morphology of HCT-116 cells is shown (Figure 4). Whereas, numerous cells in the control group had an irregular polygon attached to the wall and an integrated cytoskeleton, the morphology of cells exposed to HRS (50 μM) changed slightly after treatment for 12 h, with obvious reductions in cell number after 24 h. Shrinkage and detachment of cells were evident when the concentration was increased to 90 μM. A significant decrease in cell numbers and greater subversive morphology changes with 130 μM; specifically, chromatin condensation, loss of nuclear construction and formation of apoptosis bodies appeared. The morphology and number of HEK293T cells were not effected after treatment with 0, 50, 90, or 130 μM HRS (Supplementary Figure S1).

### Effects of HRS on the HCT-116 cell cycle

**Figure 5:**
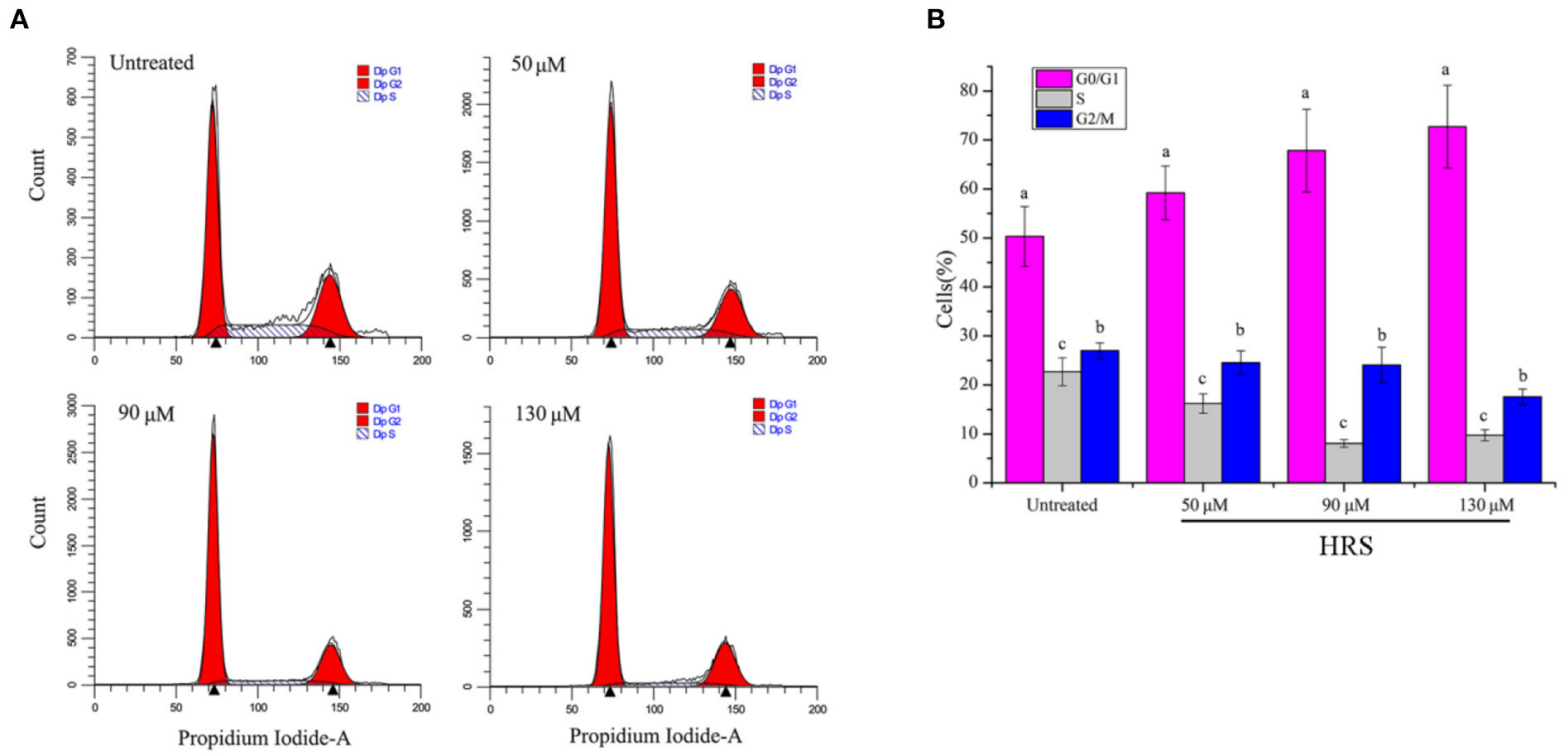
Hydroxy-γ-sanshool (HRS) induced cell cycle arrest at G1 phase in HCT-116 cell lines. **(A)** Cells in various cell cycle stages after HRS-treatment. **(B)** Percentages of cells in various cell cycle stages after treatment with HRS for 24 h. ^a−*c*^Within a concentration, columns without a common superscript differed (*p* < 0.05).

Treatment with HRS induced cell cycle arrest at the G1 phase; percentage of cells in various growth phases was determined (Figures 5A,B). The number of cells in the G1 phase were significantly increased (17.71, 34.86, and 44.50% for 50, 90, and 130 μM, respectively) compared to the untreated group on the HCT-116 cells. However, the proportion of cells in G0/G1, S, and G2/M phases were not observably changed for HEK293T cells after treatment with 0, 50, 90, or 130 μM HRS, indicating it could not stop or slow normal cell growth (Supplementary Figure S2).

**Figure 6:**
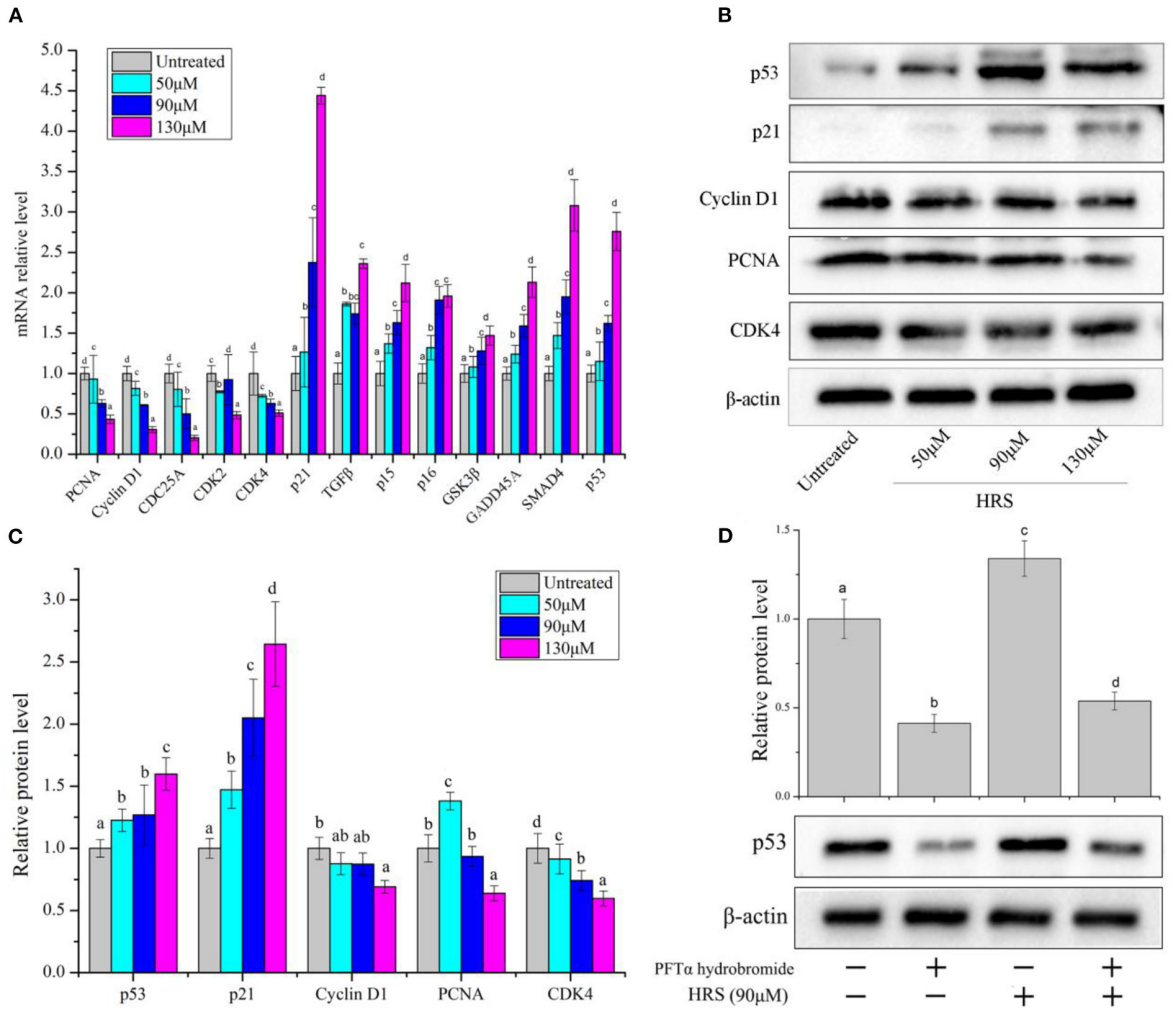
Expression of cell-cycle related protein and genes in HCT-116 cells after treatment with hydroxy-γ-sanshool (HRS). **(A)** Relative mRNA level of related genes in HCT-116 cells. **(B)** Western blot of effects on HRS on cell-cycle related proteins in HCT-116 cells. **(C)** Levels of cell-cycle related proteins. **(D)** Protein level of p53 in HCT-116 cells, with or without a p53 inhibitor (PFTα hydrobromide). HCT-116 cells were treated with HRS at designated concentrations and compared to untreated cells. All data were expressed as means ± SD for three replicated experiments. ^a−*d*^Within a cluster, columns without a common superscript differed (*p* < 0.05).

We further investigated the molecular regulation mechanism with real-time PCR and western blot analysis in HCT-116 cells treated with HRS. Relative mRNA expression of p21, TGFβ, p15, p16, GSK3β, GADD45A, SMAD4, and p53 were dose-dependently up-regulated, whereas PCNA, CyclinD1, CDC25A, CDK2, and CDK4 were significantly down-regulated (Figure 6A).

Based on the above analyses, protein expressions of selected genes (p53, p21, PCNA, CDK4, and cyclinD1) were evaluated (Figures 6B,C). Protein levels of CDK4, cyclin D1, and PCNA in treated cells were decreased; however, p53 and p21 were increased with exposure to HRS for 24 h. The p53/p21 pathway is essential in cell cycle arrest at the G1 phase. P53 can activate p21, which in return inhibits the activity of CDK4 and cyclin D1 (21). Subsequently, the PFTα hydrobromide (p53 inhibitor) remarkably decreased the HRS-induced increment of p53 (Figure 6D). Based on these results, it was suggested that the growth of HCT-116 cells was suppressed mainly through arresting cell cycle in the G1 phase, increasing the protein levels of p53 and p21, and down-regulating CDK4, cyclinD1, and PCNA.

### Effects of HRS on HCT-116 cell apoptosis

**Figure 7:**
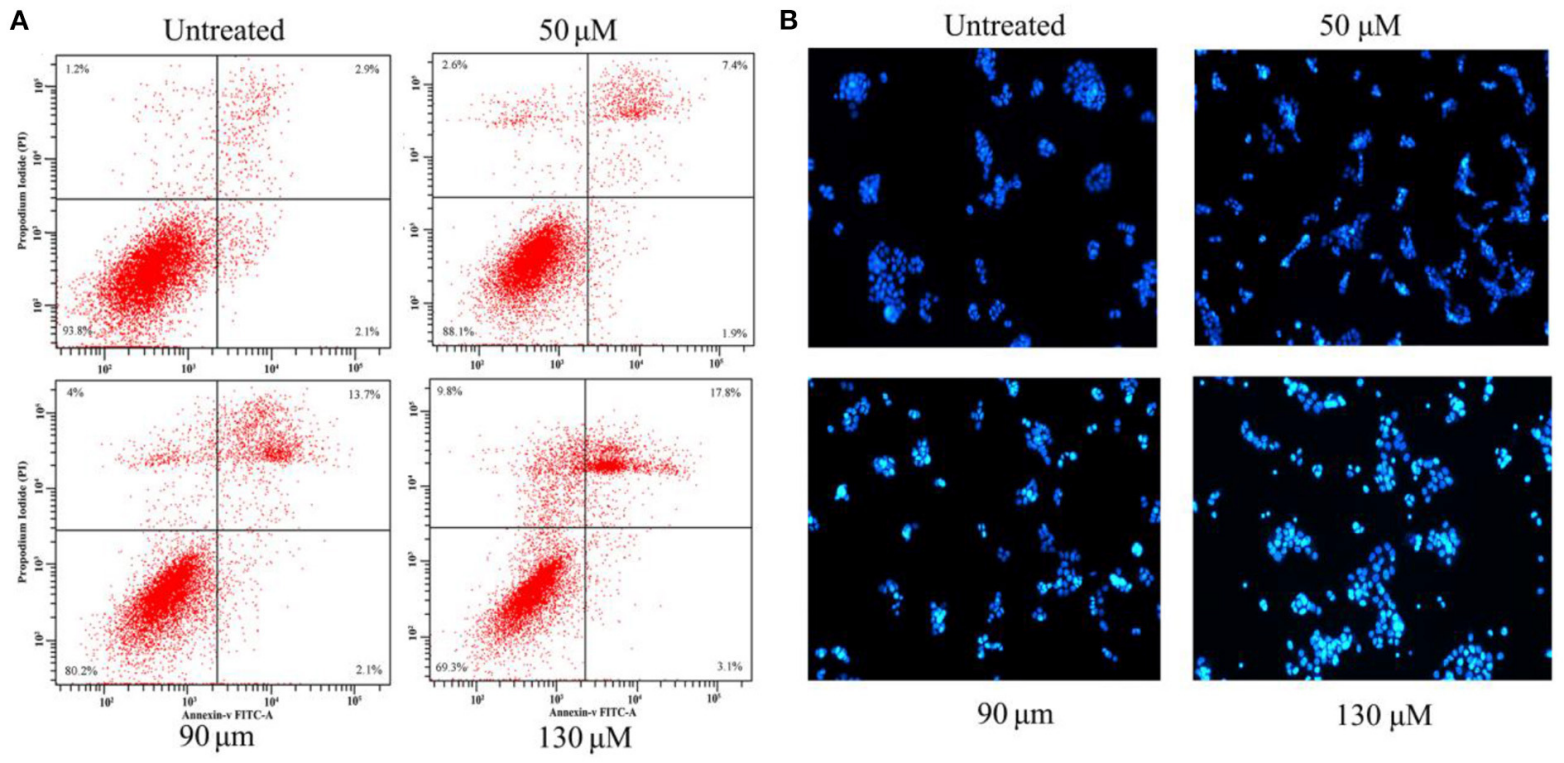
**(A)** Apoptosis in HCT-116 cells treated with various concentrations of hydroxy-γ-sanshool (HRS) for 24 h. The effect of HRS on apoptosis was assessed by flow cytometry after staining with Annexin V-PI. **(B)** Morphologic examination of treated HCT-116 cells stained with Hoechst 33342.

To confirm whether the growth inhibitory effect of HRS was due to programmed cell death, Hoechst 33342 staining and flow cytometry were used. There were 6.2, 11.9, 19.8, and 30.7% apoptotic cells after treatment with 0, 50, 90, and 130 μM HRS, respectively (Figure 7A). In addition, the apoptotic cells were detected by Hoechst 33342 staining (Figure 7B). The apoptotic cells containing apoptotic features such as nuclear shrinkage and chromatin condensation were observed, the apoptotic cell number was increased compared to the control group, and the nuclei were generally fragmented and stained more intensely following exposure to 50, 90, or 130 μM. Moreover, the edges of the nucleus were less clearly defined after treatment with 130 μM HRS. These findings suggested that HRS could induce apoptosis and necrosis of HCT-116 cells. Moreover, the nuclei of HEK293T cells appeared round and evenly stained after treatment with 0, 50, 90, and 130 μM HRS, which indicated that the apoptosis of mornal cell could not be affected by HRS (Supplementary Figure S3).

### Effects of HRS on the mitochondrial pathway

**Figure 8:**
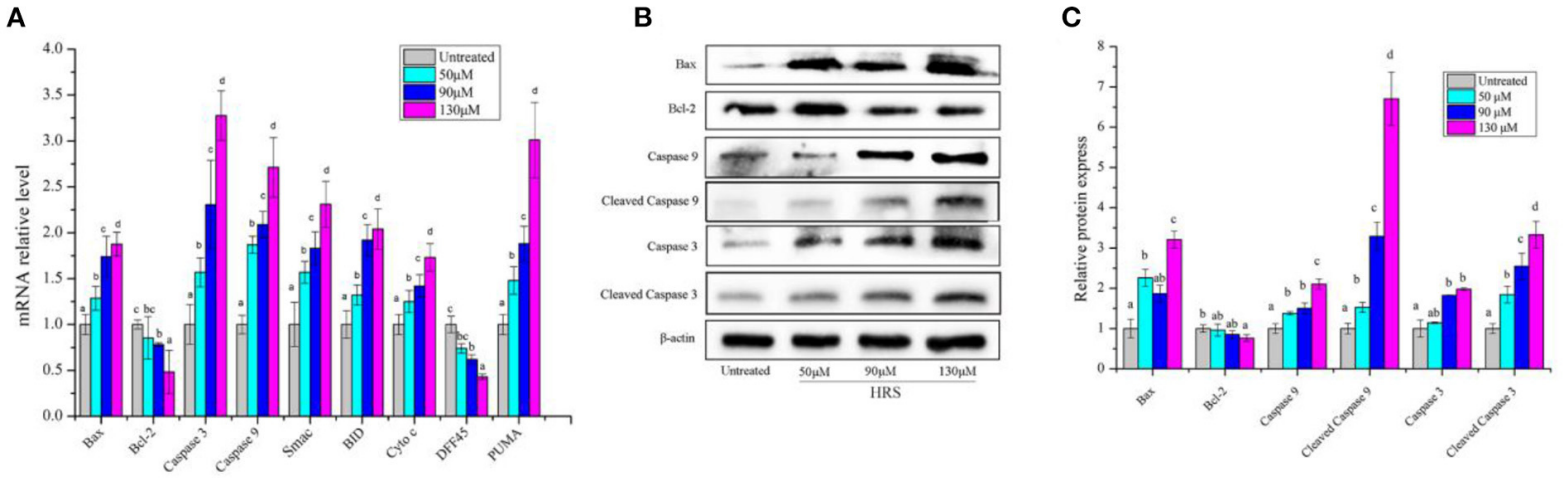
The mRNA and protein expressions of mitochondrial pathway in HCT-116 cells treated with hydroxy-γ-sanshool (HRS). **(A)** The mRNA level of mitochondrial pathway related genes. **(B)** Effects of HRS on related proteins in HCT-116 cells. **(C)** Levels of mitochondrial pathway related proteins. HCT-116 cells were treated with HRS at designated concentrations and compared to untreated cells. All data were expressed as means ± SD from three replicated experiments. ^a−d^Within a cluster, columns without a common superscript differed (*p* < 0.05).

There are two common initiation pathways (mitochondrial and death receptor) that lead to the execution of apoptosis (22). In terms of the mitochondrial pathway, expression levels of Bax, Bcl-2, Caspase 3, Caspase 9, Smac, Bid, Cyt c, DFF45, and PUMA were assessed by RT-PCR. The mRNA levels of Bax, Caspase 3, Caspase 9, Smac, Bid, Cyt c, and PUMA were increased by HRS except for Bcl-2 and DFF45 (Figure 8A). Similarly, expression of pivotal proteins in the mitochondrial pathway was further investigated. Up-regulation of protein levels of Bax, Caspase 3, Cleaved Caspase 3, Caspase 9, and Cleaved Caspase 9 and down-regulation of Bcl-2 confirmed results of RT-PCR (Figures 8B,C). These findings suggested that the mitochondrial pathway has an essential role in HRS-induced apoptosis and led us to further focus on the receptor-mediated pathway.

### Effects of HRS on death receptor pathway

**Figure 9:**
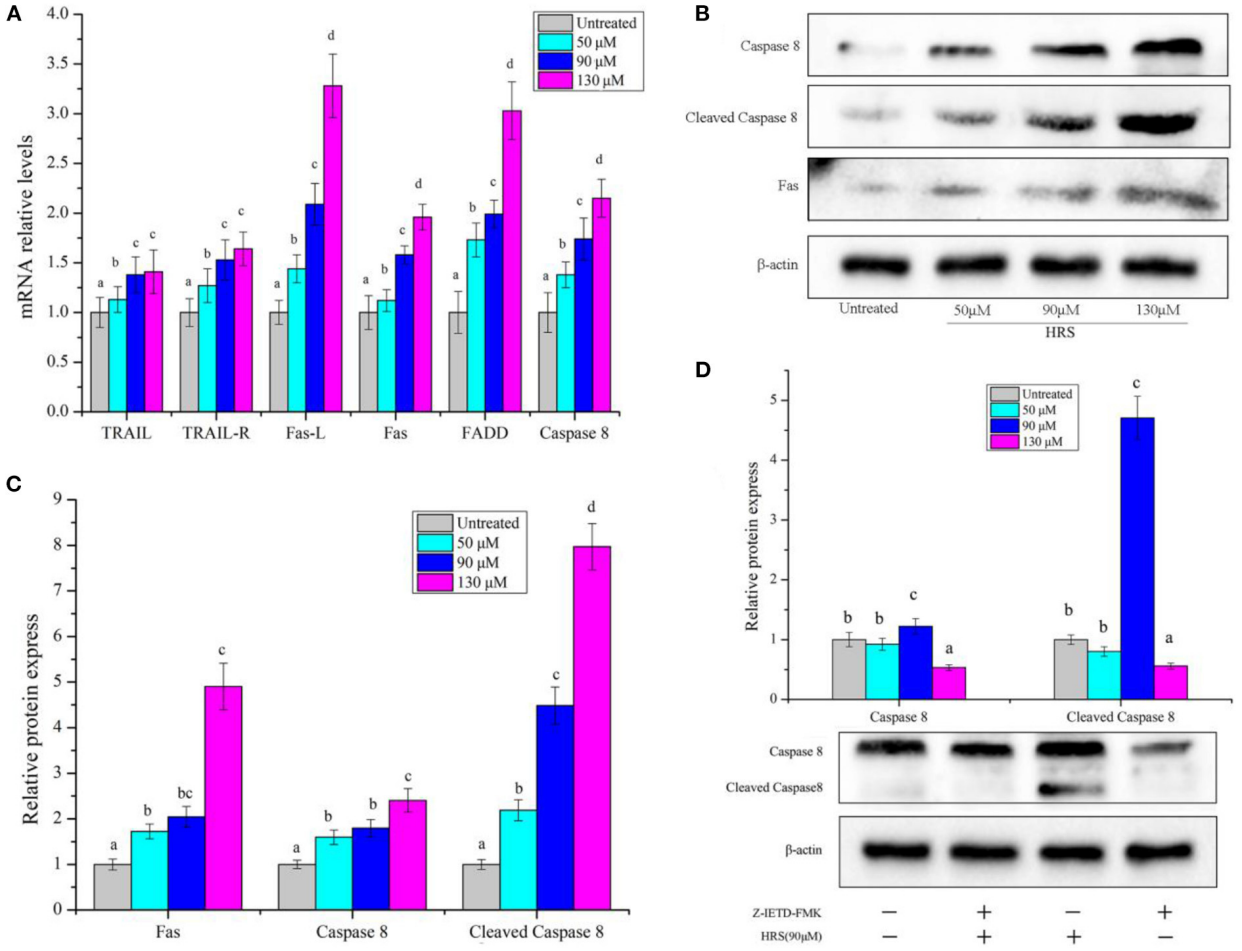
The mRNA level of death receptor pathway genes and protein in HCT-116 cells treated with hydroxy-γ-sanshool (HRS). **(A)** The mRNA level of genes in HCT-116 cells. **(B)** Western blot analyses of related genes. **(C)** Relative protein expression of related genes. **(D)** Protein level of Caspase 8 in HCT-116 cells, with or without an inhibitor (Z-IETD-FMK). HCT-116 cells were treated with HRS at designated concentrations and compared to untreated cells. All data were expressed as means ± SD from three replicated experiments. ^a−d^Within a cluster, columns without a common superscript differed (*p* < 0.05).

In the death receptor pathway, mRNA levels of several genes (TRAIL, TRAIL-R, Fas-L, Fas, FADD, and Caspase 8) (Figure 9A) and protein levels of Fas, Caspase 8, and Cleaved Caspase 8 (Figures 9B,C) were increased by HRS after treatment for 24 h. Furthermore, a Caspase 8 inhibitor (Z-IETD-FMK) was used to confirm the role of Caspase 8 and Cleaved Caspase 8 in the death receptor pathway in HRS-induced apoptosis (Figure 9D). Pretreatment with Z-IETD-FMK significantly decreased Caspase 8 and Cleaved Caspase 8 protein expression, which was reduced to some extent with addition of HRS, implicating involvement of the death receptor pathway.

## Discussion

Consumption of various natural products may prevent cancer as they contain various phytochemicals. Prickly ash belongs to the Rutaceae family and is widely distributed in the tropical and subtropical areas of Asia and America (23). It is listed in the Pharmacopeia, with >30 prescriptions for the treatment of dyspepsia, vomiting, toothache, ascariasis, eczema, abdominal pain, etc. (24). Sanshools are the primary compounds of polyunsaturated amides, regarded as the critical constituents in the majority of the biological activities of prickly ash. In several studies, sanshool extracts had noticeable anticancer effects in human cancer cell lines DLD-1, HepG2, and Caco-2 (12). Furthermore, according to the modular variation, >10 types of sanshools with various structures have been identified. Sanshools structure is commonly allocated into three separate parts: vanillyl group, amide bond, and fatty chain. However, comparison of anticancer activity sanshools as a function of their structural distinctions is not well characterized. Therefore, the current study is an attempt to compare inhibitory effects of sanshools (HAS, HBS, and HRS) on a colon cancer cell line (HCT-116) and investigate the possible mode of apoptosis induction.

In our studies, the three sanshools had distinctly different inhibition potency (IC_50_ were 215.06, 114.63, and 88.01 μM, respectively), despite all being isolated from the same species of prickly ash. The inhibition effect of HBS was superior to HAS, attributed to the difference between *cis*- and *trans*-isomer (25). In contrast, the *cis*-isomer HAS was superior to its *trans*-isomer HBS on inhibiting a neurofibromatosis type 1 (*NF*_1_)-defective tumor cell (26). Perhaps these opposing results were due to the differences in study cell lines and their pathways (27). The IC_50_ of HRS was lowest compared to HAS and HBS, attributed to the quantity and location of *carbon-carbon double bonds* in the sanshool molecule (28). Compounds with more *carbon-carbon double bonds* are readily peroxidized, yielding more and different peroxides that induce cell apoptosis (28). Furthermore, variations in locations of *carbon-carbon double bonds* affect functional groups and alter various biological activities (29). Regardless, sanshool had low toxicity and no significant inhibitory effect on normal cells, indicating high selectivity toward cancer cells.

The cell cycle is a collection of ordered processes that coordinate DNA replication and cell division. However, a human cancer cell has deregulation of the cell cycle and unscheduled proliferation. The present study produced convincing evidence that HRS induced cell cycle arrest in the G1 phase and inhibited proliferation of HCT-116 cells, consistent with previous results (16). Although effects of HRS-induced cell cycle arrest were indicated by these results, underlying mechanisms remain to be elaborated. In present study, HRS treatment of HCT-116 cells induced dose-responsive cell-cycle arrest by decreasing mRNA and protein expression levels of CDK4 and CyclinD1. Expression of Cyclin D1 and CDK4 (16) were investigated because they are the key genes of the G1 phase and form active cyclinD1/CDK4 complexes (30), ultimately initiating G1-S transition (31). In addition, HRS increased protein and mRNA levels of p53 and p21, while decreasing expression of PCNA in HCT-116 cells, indicating that p53 may be the key factor affecting the cell cycle. P53 controls CDk4 activity and PCNA-dependent DNA replication to alter cell cycle progression *via* regulation of protein p21.

Next, we further determined the role of p53 in the cell cycle arrest process. The tumor suppressor p53 functions is a gene-specific transcription factor (32). Two physiological outcomes of expression of p53 in tumor cells are G1 arrest and apoptosis, whereas, the trigger of G1 arrest by p53 is much better than its ability to induce apoptosis. P53 induces the expression of p21, an inhibitor of cyclin-dependent kinases 2, 3, 4, and 6 (33). Thus, a G1 arrest can result from the p53-induced expression of p21 and we choose p53 as the key protein to research its effect on cell cycle regulation. Pifithrin-alpha (PFT) a useful p53 inhibitor (34), decreased protein expression of p53 and suppressed the HRS-induced cell cycle arrest in HCT-116 cells. Therefore, HRS-induced arrest of the cell cycle in the G1 phase was attributed to increased expression of p53 in HCT-116 cells. Similarly, transforming acidic coiled-coil (TACC) depletion-induced G1 arrest and cell death was reduced in cells, indicating that induction of G1 arrest and cell death require p53 (35). In addition, inhibiting expression of p53 protein in SGC7901 cells blocked cell arrest and cell apoptosis (34).

The extrinsic (receptor-mediated) and intrinsic (mitochondria-mediated) pathways of apoptosis are well described (22); in the present study, HRS initiated apoptosis of HCT-116 cells through both pathways. The mitochondrial pathway is initiated by internal stimuli and regulated by Bcl-2 family proteins, including anti-apoptotic proteins (Bcl-2, Bcl-XL, and Bcl-W) and pro-apoptotic proteins (Bax, Bid, etc.) (36). In this study, HRS improved the mRNA expression of Bax, Caspase 3, Caspase 9, BID, Cyt c, and PUMA except for Bcl-2 (Figure 8). These anti- and pro-apoptotic proteins may regulate mitochondrial release of Cyt-c that in turn activates Caspase 9 and 3 (37), the molecular “scissors” of apoptosis. Protein expression of Bax, Caspase 9, Cleaved Caspase 9, Bcl-2, Caspase 3, and Cleaved Caspase 3 was consistent with changes in mRNA, indicating that the Bcl-2 family of proteins was involved in HRS-induced apoptosis. Furthermore, in our study, HRS increased relative mRNA expression of Smac, and decreased the level of DFF45. Smac improves caspase activation by combining inhibitors of apoptosis proteins. Ultimately, Caspase 3-mediated cleavage of DFF45 releases DFF40, leading to chromatin condensation and triggering cell death (38). Based on these findings, we inferred that the mitochondria-dependent apoptotic pathway was activated by HRS in HCT-116 cells. Similarly, sanshools extracted from the leaf of *Zanthoxylum* also induced apoptosis of Jurkat cells through a mitochondria-dependent pathway (39).

The death receptor pathway, the key executor of apoptosis, is another important signaling mechanism in anticancer therapy. Genes such as TRAIL, TRAIL-R1, Fas-L, Fas, FADD, and Caspase 8, and protein levels of Fas, Caspase 8, and Cleaved Caspase 8 were all up regulated by HRS (Figure 9), implicating initiation of death-receptor mediated pathway of apoptosis. Members of the death receptor family, Fas and TRAILR1 containing the death domain (DD) integrated with FasL and TRAIL, respectively, and once bound, activated Caspase 8 which initiates the pivotal pro-apoptotic protein caspase 3 (40), followed by programmed cell death. This is apparently the first report that Caspase 8 and 3 were activated and Fas was significantly increased in HRS-treated HCT-116 cells. Caspase 8 is regarded as the primary mediator of extrinsic apoptosis (41). It belongs to the caspase family of proteases and plays a key role in the regulation of programmed cell death during normal development (41). Extrinsic apoptosis relies on the formation of a death-inducing signaling complex (DISC), which always includes Caspase 8 (42). In addition, Caspase-8 can cleave the protein BID and cleaved BID (cBID) then activates the effector proteins BAX and BAK, which mediate mitochondrial outer membrane permeabilization, which promote the formation of the socalled “apoptosome”, and the activation of caspases that orchestrate the death of the cell (43). Therefore, we explored whether HRS-induced extrinsic apoptosis through activation of Caspase 8. Pretreatment of HCT-116 with Z-IETD-FMK (Caspase 8 inhibitor) reduced Caspase 8 protein concentration and abolished HRS-induced death receptor apoptosis. Therefore, we concluded that HRS-induced death receptor apoptosis was mediated *via* activation of Caspase 8, consistent with previous studies (44). Following treatment with Z-IETD-FMK, the number of apoptotic hepatocellular carcinoma cells decreased, and release of cytochrome c into the cytoplasm was prevented (45). Furthermore, Cadmium (Cd)-induced apoptotic morphological changes and increase of domain death agonist (tBID/BID) were mitigated by a Caspase 8 inhibitor (46).

## Conclusions

**Figure 10:**
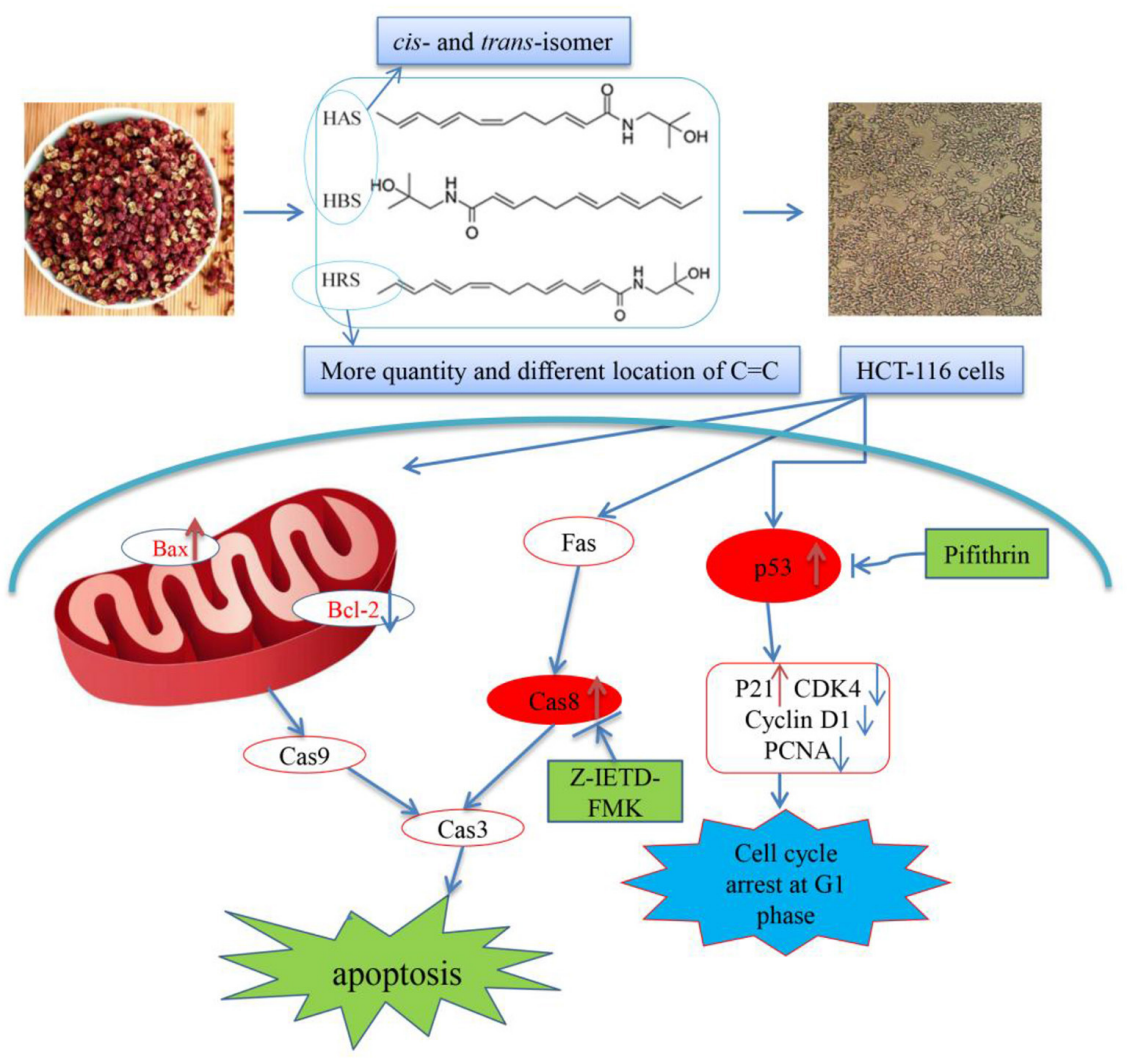
Proposed mechanism of the induced apoptotic effects of hydroxy-γ-sanshool (HRS) on HCT-116 cells.

In this study, HRS, an important active component of sanshools, was isolated, identified and used to explore an inhibition effect on HCT-116 cells proliferation. Clearly, HRS induced cell cycle arrest at the G1 phase and promoted apoptosis in dose- and time-dependent manners. Activation of p53 appeared to be involved in arresting the cell cycle in the G_1_ phase, and both the death receptor and mitochondrial pathway were involved in HRS-induced apoptosis. Therefore, we concluded that HRS has potential as a functional food. Based on the current outcomes, a flow diagram accounting for the anti-cancer effects of HRS, including induction of cell cycle arrest and apoptosis, has been prepared (Figure 10). Nevertheless, the inhibition effect needs further study to better understand underlying mechanisms.

## Data availability statement

The raw data supporting the conclusions of this article will be made available by the authors, without undue reservation.

## Author contributions

CZ: conceptualization and writing-original draft preparation. TL: methodology and software. FX: visualization. FX and LZ: investigation. SA-n: data curation. LX: supervision. All authors contributed to the article and approved the submitted version.

## Funding

This work was supported by the Science and Technology Department of Guizhou Province [2020]1Y039 and Guizhou Science and Technology Cooperation Platform Talent Project ([2019] No. 5201).

## Conflict of interest

The authors declare that the research was conducted in the absence of any commercial or financial relationships that could be construed as a potential conflict of interest.

## Publisher's note

All claims expressed in this article are solely those of the authors and do not necessarily represent those of their affiliated organizations, or those of the publisher, the editors and the reviewers. Any product that may be evaluated in this article, or claim that may be made by its manufacturer, is not guaranteed or endorsed by the publisher.
