# Supplementary material for: Hydroxy-γ-sanshool from Zanthoxylum bungeanum (prickly ash) induces apoptosis of human colorectal cancer cell by activating P53 and Caspase 8
Source: Front Nutr. 2022 Aug 1;9:914638. doi: 10.3389/fnut.2022.914638 (PMC9376619; doi:10.3389/fnut.2022.914638)

**Figure S1.** Effects of hydroxy-γ-sanshool (HRS) on morphological changed in HEK293T cells. The original magnificantion was ×100.


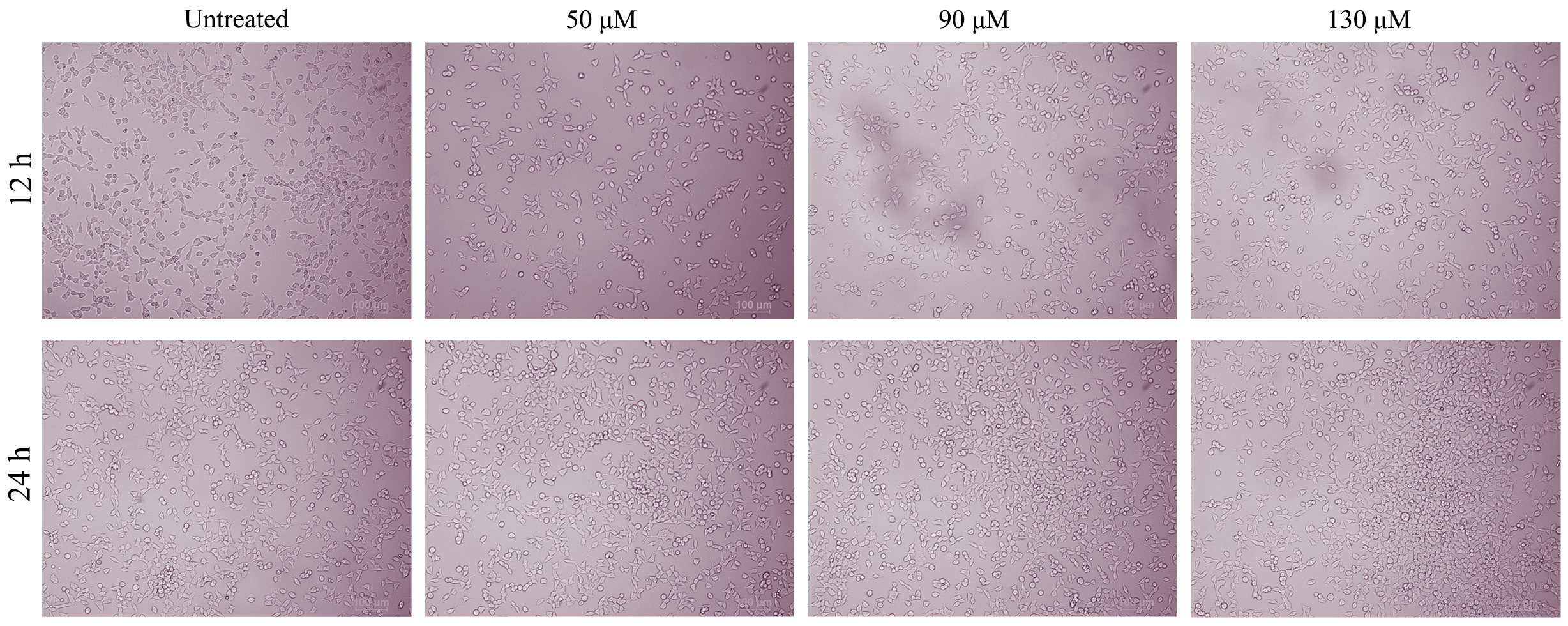


**Figure S2.** Effects of hydroxy-γ-sanshool (HRS) on cell phases were analysed in HEK293T cells using flow cytometry.


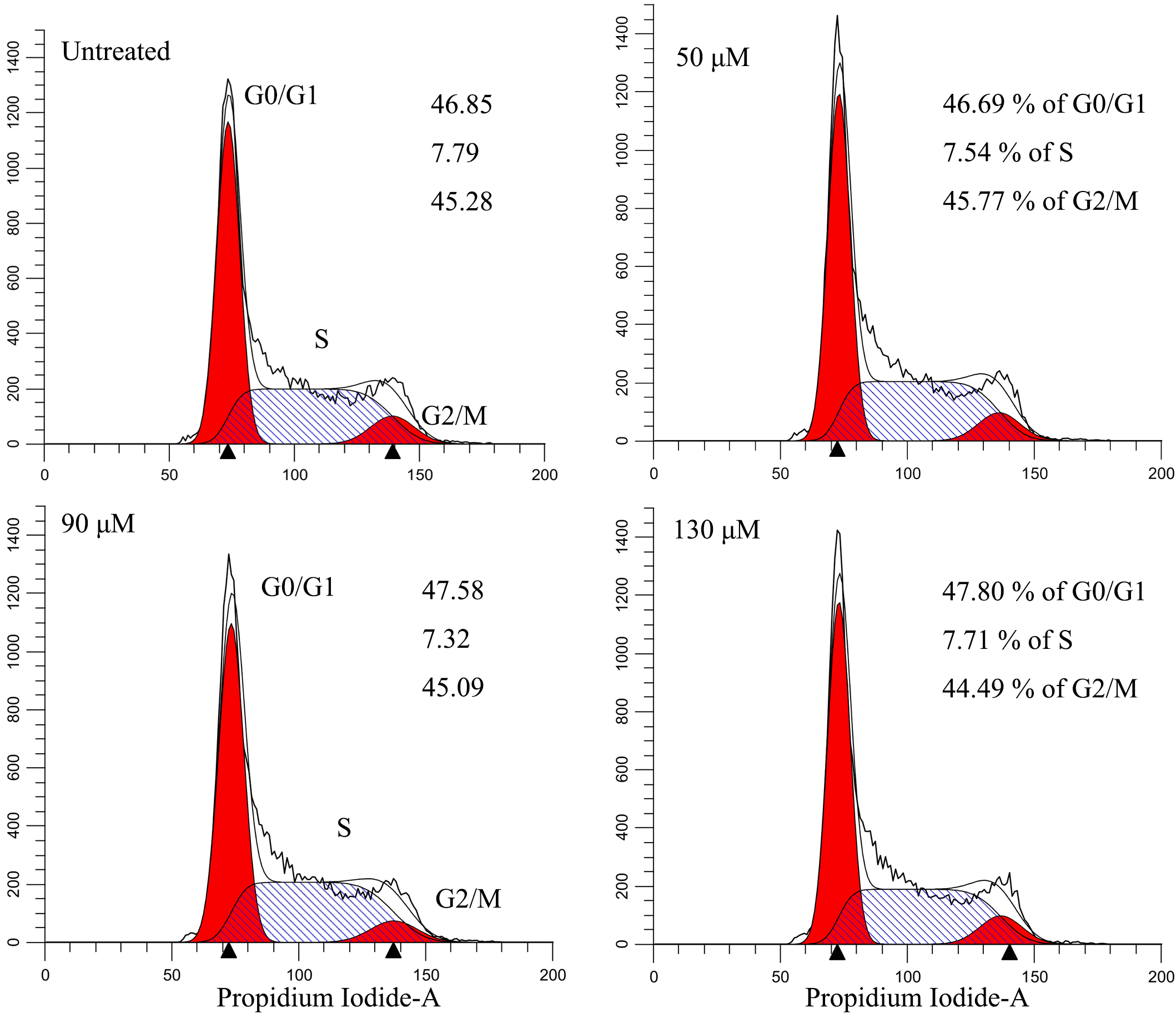


**Figure S3.** Effects of hydroxy-γ-sanshool (HRS) on cell apoptosis was detected by Hoechst 33342 staining assay in HEK293T cells. The original magnificantion was ×200.


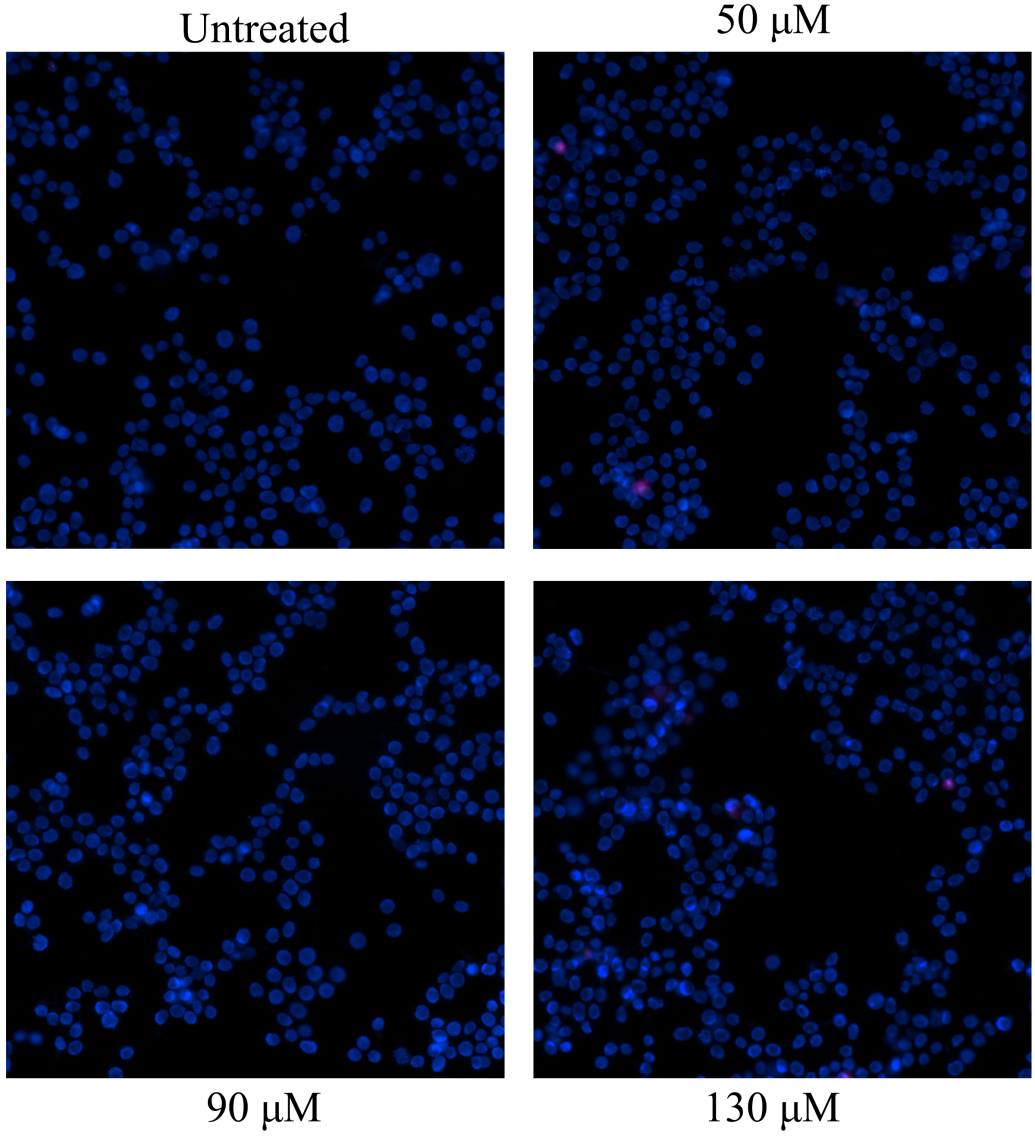

Supplement: Supplementary file 1 [file Data_Sheet_1.DOCX]
